# Identification of Hybrid Sturgeon (*Acipenser baerii* × *Acipenser schrenckii*) from Their Parents Using Germplasm

**DOI:** 10.3390/ani15070907

**Published:** 2025-03-21

**Authors:** Shiyong Yang, Zhongmeng Zhao, Zihan Xu, Ya Liu, Menghao Jiang, Lin Fu, Jin Zhang, Zhaoxin Jing, Xiaojian Pang, Wuyuntana Shao, Chaoyang Zhang, Yunkun Li, Xiaogang Du, Jiayun Wu

**Affiliations:** 1 Department of Aquaculture, Sichuan Agricultural University, Chengdu 611130, China; yangshiyong@sicau.edu.cn (S.Y.);; 2 The Fishery Research Institute, Sichuan Academy of Agricultural Sciences, Chengdu 611731, China; 3 Center for Conservation and Utilization of Rare and Endemic Fishes in Sichuan, Chongzhou 611230, China; 4 College of Life Sciences, Sichuan Agricultural University, Ya’an 625000, China

**Keywords:** sturgeons, hybrids, identification, mitochondrial, microsatellites

## Abstract

The hybrid sturgeon *Acipenser baerii* × *A. schrenckii* is the most widely cultured commercial sturgeon in China. However, its morphological similarity to the parental species frequently leads to misuse of germplasm in the breeding process, resulting in a decline in the quality of the sturgeon production. In this study, we have developed a protocol by using mitochondrial DNA barcoding and microsatellite locus analysis for the accurate identification of sturgeon species. Genetic distance and phylogenetic analysis based on the mitochondrial COI segment showed that *A. baerii* exhibited the closest genetic relationship with orthogonal individuals *A. baerii* (♀) × *A. schrenckii* (♂). Conversely, *A. schrenckii* displayed the highest genetic similarity with reciprocal individuals *A. schrenckii* (♀) × *A. baerii* (♂). Additionally, genetic structure analysis and factor correlation analysis (FCA) were conducted using six microsatellite loci among 100 samples, including eight species and two hybrid sturgeon. The results showed that all samples, encompassing both hybrid sturgeon (*A. baerii* × *A. schrenckii*) and their parental species, were accurately grouped into ten clusters, thereby validating the precision of this species assignment method.

## Simple Summary

The morphological similarity of the hybrid *Acipenser baerii* × *A. schrenckii* to their parental species often results in misuse of germplasm during the breeding process, leading to a decline in the quality of sturgeon production. In this study, we assembled samples from eight pure sturgeon species and two hybrid sturgeons, and conducted accurate discrimination using mitochondrial DNA barcoding and microsatellite locus analysis. The findings reveal clear genetic differentiation among the hybrid sturgeon (*A. baerii* × *A. schrenckii*) and their parental species, effectively distinguishing them from the other sturgeon species included in the analysis. Notably, the hybrid sturgeons *A. baerii* (♀)× *A. schrenckii* (♂) and *A. schrenckii* (♀) × *A. baerii* (♂) exhibited the closest genetic relationship with their respective maternal parents, *A. baerii* and *A. schrenckii*. This study provides a highly accurate methodology for the identification of *A. baerii*, *A. schrenckii* and their hybrids, which is beneficial for ensuring accurate germplasm utilization in hybrid sturgeon breeding processes and enhancing their economic value.

## 1. Introduction

Sturgeon species are known as fish of economic importance. In addition to being food for humans, they are also used in healthcare, the leather industry, and ornament manufacturing. Caviar, made from sturgeon eggs and vulgarly called “black gold”, is a precious food with an extremely high price and a long history in the international market [1]. Since the latter half of the twentieth century, over-fishing combined with environmental pollution and construction for water conservancy have resulted in a sharp decline in the population of wild sturgeon throughout the world [2,3]. All sturgeon species have been protected under the Convention on International Trade in Endangered Species of Wild Fauna and Flora (CITES) in China since 1998. Sturgeon farming has emerged to meet the increasing demand for sturgeon meat and caviar products. However, with the rapid development of the culture industry, breeding issues have become increasingly prominent. For example, the quality of purebred sturgeon has generally declined, their stress resistance has significantly decreased, and the fertilization rate and reproductive efficiency of purebred sturgeon eggs have progressively declined. To address these issues, the application of crossbreeding technology to cultivate new hybrid species with stronger adaptability, higher resistance to disease, and faster growth rates has emerged as a feasible solution [1,4,5]. Therefore, hybrid sturgeons have become the most widely cultured commercial sturgeon in China, including the hybrid of *Acipenser schrenckii* × *Huso dauricus* for caviar production and the hybrid of *A. baerii* × *A. schrenckii* for commercial fish production [6]. While hybrid breeding has improved the survival rate of cultured sturgeons and generated economic benefits, it has also introduced adverse effects. Specifically, indiscriminate hybridization often results in low-quality seedlings, and the proliferation of hybrid varieties poses threats to wild resources and unpredictable ecological consequences. Therefore, establishing a clear pedigree map to obtain high-quality seedlings is crucial for the development of sturgeon aquaculture and the accurate identification of hybrids.

*Acipenser baerii* and *A. schrenckii* are two prominent sturgeon species cultured in China. *Acipenser schrenckii*, distinguished by its high growth rate, is endemically distributed in the Heilongjiang (Amur River) watershed and holds significant economic importance in China’s aquaculture industry [7]. However, its susceptibility to disease, intolerance to transportation stress, and excessive dependence on live bait often hinder its adaptability and result in high mortality rates. Conversely, *A*. *baerii* primarily inhabits Central Asia and Eastern Europe, with small population inhabiting the Irtysh River System in China [8]. Although exhibiting a slower growth rate compared to *A*. *schrenckii*, *A. baerii* demonstrates strong resistance to disease and tolerance to transportation stress [1]. Naturally, hybridization between *A*. *baerii* and *A. schrenckii* is uncommon due to their substantial habitat differences and limited population sizes [7,8]. The situation changed in sturgeon breeding facilities. In 2007, the successful crossbreeding of *A. baerii* and *A. schrenckii* was achieved. The resulting hybrids displayed enhanced adaptability, accelerated growth, superior disease resistance, and higher survival rate during transportation compared to their parental species. These advantageous traits have made the hybrid sturgeon the most extensively cultivated and produced sturgeon in China.

However, the morphological similarity among *A. baerii*, *A. schrenckii*, and their hybrids can cause confusion in production processes, ultimately resulting in economic losses for farmers. Currently, a variety of genetic marker-based methods are available for identifying fish hybrids, including PCR–restriction fragment length polymorphism (RFLP), random amplified polymorphic DNA (RAPD), restriction site-associated DNA sequencing (ddRAD), simple sequence repeats (SSRs), DNA barcode, single-nucleotide polymorphism (SNP), and whole-genome resequencing [9,10,11,12,13,14,15]. However, RFLP and certain other methods have demonstrated poor reproducibility and are no longer preferred. Moreover, next-generation sequencing methods may not be easily manageable for routine analyses by fish farmers and breeders. Among these techniques, DNA barcode markers and SSRs are the most widely used due to their high resolution, rapid detection capabilities, and ease of operation [16]. The aim of this study was to accurately identify *A. baerii*, *A. schrenckii*, and their hybrids, thereby providing theoretical guidance for the identification of artificially cultivated sturgeon species.

## 2. Materials and Methods

### 2.1. Fish Sampling

Caudal fins were collected from 10 individuals of *A. baerii*, 10 of *A. schrenckii*, 15 hybrids of *A. baerii* (♀) × *A. schrenckii* (♂), 15 hybrid individuals of *A. schrenckii* (♀) × *A. baerii* (♂), 10 of *Huso dauricus*, 10 of *H. huso*, and 10 of *A. gueldenstaedtii* from Sichuan Runzhao Fisheries Co., Ltd. (Chongzhou, China) in 2018. Additionally, caudal fins were collected from 10 individuals of *A. sinensis*, 10 of *A. dabryanus*, and 10 of *A. ruthenus* from Xinxing Aquatic Products Co., Ltd. (Chengdu, China) in 2012. All collected sturgeon fins were preserved in absolute ethanol for total DNA extraction. Total genomic DNA was extracted using the Genomic DNA Extraction Kit (Tiangen, Shanghai, China). All sample collections adhered to Chinese regulations for the implementation of the protection of terrestrial wild animals (State Council Decree [1992] No. 13).

### 2.2. Sequencing and Genotyping

Universal cytochrome c oxidase subunit I (COI) primers specific for sturgeon species (FR1d: CAGGAAACAGCTATGACCACCTCAGGGTGTCCGAARAAYCARAAa; FishR2: CAGGAAACAGCTATGACCACTTCAGGGTGACCGAAGAATCAGAAa; FishF2: TGTAAAACGACGGCCAGTCGACTAATCATAAAGATATCGGCACa; VF2:TGTAAAACGACGGCCAGTCAACCAACCACAAAGACATTGGCACa) were used for PCR amplification of *A. baerii*, *A. schrenckii*, and their hybrid progeny [17]. All PCR reactions were performed in a total volume of 25 µL, containing 1 µL 2.5 mM dNTPs, 2 µL 1.5 mM MgCl2, 2.5 µL 10 × reaction buffer, 0.3 µL Taq DNA polymerase (2.5 U µL–1) (Tiangen), 0.5 µL 25 pM each primer, and 30–50 ng genomic DNA. All amplifications were performed under the following conditions: 3 min at 94 °C; 35 cycles at 94 °C for 30 s, 56 °C for 30 s, and 72 °C for 90 s; and a 10 min extension at 72 °C. After amplification, the PCR products were purified using a universal DNA purification Kit DP214-02 (Tiangen) and sequenced directly on an ABI 3730XL automatic sequencer (Invitrogen Corporation, Shanghai, China). The COI sequences of *H. dauricus* (KC578834), *H. huso* (FJ809715), *A. sinensis* (KP218536), *A. dabryanus* (KP218556), *A. gueldenstaedti* (KC500088), *A. transmontanus* (EU523887), *A. fulvescens* (EU524393), *A. stellatus* (KC500127), *A. ruthenus* (KC578827), *Scaphirhynchus albus* (AP004354), *S. platorynchus* (JN028407), *Polyodon spathula* (AP004353), and *Psephurus gladius* (AY571339) were downloaded from GenBank and analyzed together with the COI sequences obtained from the sturgeon samples in this study.

For the genotyping experiments, eight species and two hybrids of sturgeons were analyzed using six pairs of microsatellite primers (Table 1). A total of twenty-four microsatellite primers were synthesized and subjected to amplification, with six microsatellite loci exhibiting clear and distinguishable polymorphisms among the sturgeon species. The primers Ab3, Ab5, and As1 were specifically designed for this study, whereas LS19, HLJSX7, and HLJSX30 were previously reported for *A. schrenckii* by Hu et al. [18]. The primer pairs were fluorescently labeled with FAM. PCR amplification was performed under the following conditions: preheating at 94 °C for 4 min; denaturing at 94 °C for 40 s, annealing at 50–58 °C for 30 s, and elongation at 72 °C for 40 s for 35 cycles; and extension at 72 °C for 10 min. Amplifications were carried out in 15 μL reaction volumes containing 10 ng template DNA, 0.5 μL each primer, 6 μL ddH_2_O, and 7.5 μL 2× Premix Taq DNA polymerase (Takara, Dalian, China). All PCR reactions were carried out using a PTC 100 thermal cycler (MJ Research, Waltham, MA, USA). Allele sizing was carried out by automated fluorescent scanning detection in an ABI 377XL DNA sequencer (Applied Biosystems, Carlsbad, CA, USA) with software GENESCAN (v.3.7) and GENOTYPER (Applied Biosystems, v3.1), and ROX500 (MCLAB, San Francisco, CA, USA) serving as the internal lane size standard. Individual polyploidy genotypes were scored from microsatellite banding patterns observed in the electropherograms, according to the Microsatellite DNA Allele Counting Peak Ratio (MAC-PR) method described by Esselink et al. [19].

### 2.3. Statistical Analyses

The obtained COI sequences were aligned, and basic parameters including polymorphic sites, conversion sites, transitions, transversions, and insertions/deletions were calculated using MEGA (v5.0) [20]. The software DnaSP (v4.50) [21] was used to calculate genetic distances between and within groups based on the Kimura 2-parameter model. The phylogenetic tree was constructed using the Bayesian Inference (BI) method implemented in MrBayes (v.3.2) [22]. Additionally, BLAST (v.2.6.0) was used to compare the COI sequences among groups and calculate sequence similarity. The software Structure (v 2.3.2) [23] was used to infer strain composition based on microsatellite data. The Bayesian clustering approach was used in Structure (v.2.3.2) to identify the most likely number of clusters (K) as well as to assign individuals to these clusters. Ten replicate runs (burn-in period of 100,000 steps and 100,000 MCMC iterations) were performed at each value of K from 2 to 8. Individuals were assigned based on their membership coefficient. Furthermore, factorial correspondence analysis (FCA) based on multi-locus genotypes was conducted using the GENETIX (v.4.05) program to distinguish strains and identify any intermediate genotypes resulting from strain admixture. The R package POLYSAT (v.1.7-7) was used to calculate the number of alleles, allele frequency, and the Shannon–Wiener index. The Simpson index was used to characterize the levels of genetic diversity in each sturgeon strain.

## 3. Results

### 3.1. Genetic Variation Among the Hybrid Sturgeon and Their Parents

A total of 41 COI gene sequences were obtained from 15 individuals of *A. baerii*, 6 of *A. schrenckii*, 9 of orthogonal hybrid individuals, and 11 reciprocal hybrid individuals. Table 2 provides the basic information about the COI sequences of *A. schrenckii*, *A. baeri*, and their hybrids. Sequence similarities calculated with BLAST between COI segments of *A. baerii* and orthogonal hybrid individuals exceeded 99%, with comparable results observed for *A. schrenckii* and reciprocal hybrid individuals (sequence similarities: 99–100%). The slight variations observed in the mitochondrial COI genes between the hybrids and their maternal parent may be influenced by genetic introgression from the species.

### 3.2. The Hybrid Sturgeons Sepearted from Their Male Parent by Phylogenetic Analysis

**Figure 1:**
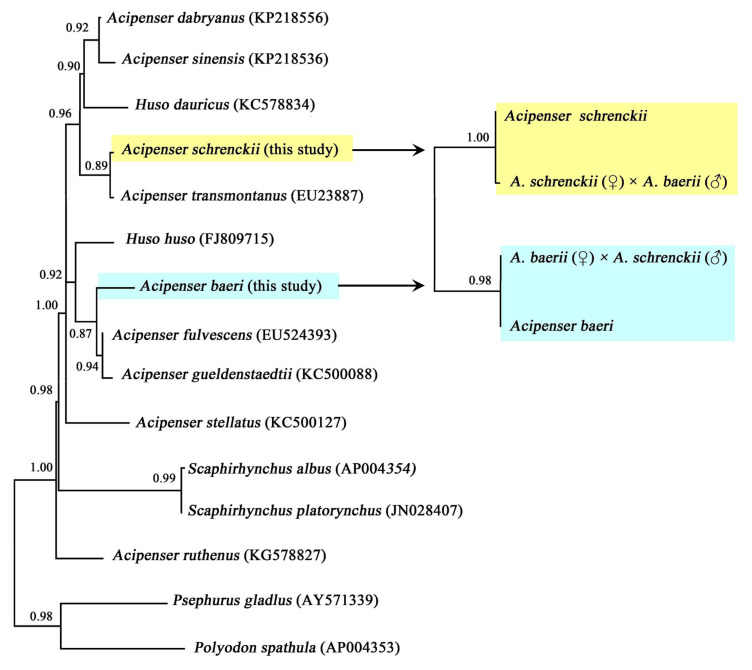
Bayesian Inference (BI) phylogenetic tree constructed for different sturgeons, and for hybrids and their parental sturgeons, based on the COI gene. Support rates are annotated on the nodes. The yellow background indicates clustering of *A. schrenckii* and its hybrids, and the green background indicates clustering of *A. baeri* and its hybrids.

The mean intra-group genetic distances of *A. baerii*, *A. schrenckii*, and their hybrid offsprings were notably low, ranging from 0.0000 to 0.0016 (Table 3), indicating a high degree of conservatism in the COI sequences of these species. Interspecific genetic distances between *A. baerii* and orthogonal hybrids, as well as between *A. schrenckii* and reciprocal hybrids, were substantially smaller (0.0000–0.0040) compared to the distances to other sturgeon species (Table 3). Fifteen individuals of *A. baerii* and six of *A. schrenckii* were clustered together. The sturgeon species *A. baeri* clustered with *A. fulvescens* and *A. gueldenstaedtii*, while *A. schrenckii* clustered with *A. transmontanus*, indicating a close genetic relationship among these species. The orthogonal hybrids clustered with *A. baerii*, whereas all reciprocal hybrids grouped with *A. schrenckii* (Figure 1).

### 3.3. The Hybrid Sturgeons Were Separated from A. sinesis by Structure Analysis

**Figure 2:**
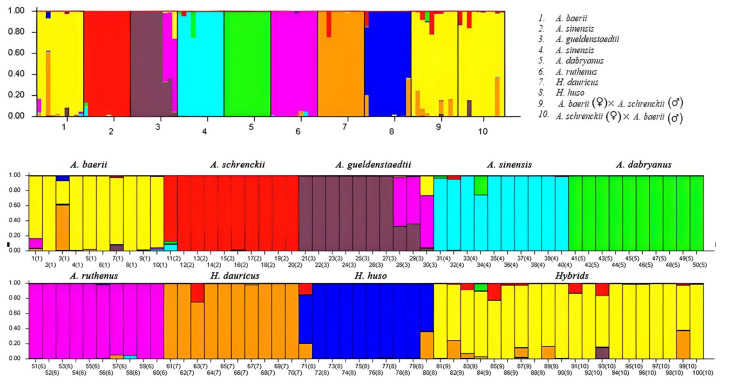
Assignment of 100 sturgeons by structure analysis based on six microsatellite loci in eight species and two hybrids of sturgeon. Histograms represent the estimated membership coefficients (Q). The composite bars represent expected hybrids. Different colors represent to different sturgeon species.

Characteristic information was collected on the total number of alleles at each locus, the allelic frequencies, and the potential private alleles, including diagnostic alleles, which ranged in frequency from 0.01 to 1 (Table 4). The structure assignment test, conducted across K values ranging from 2 to 8, confirmed the presence of 10 distinct clusters: (1) *A. baerii*; (2) *A. schrenckii*; (3) *A. gueldenstaedtii*; (4) *A. sinensis*; (5) *A. dabryanus*; (6) *A. ruthenus*; (7) *H. dauricus*; (8) *H. huso*; (9) *A. baerii* (♀) × *A. schrenckii* (♂); and (10) *A. baerii* (♂) × *A. schrenckii* (♀) (Figure 2). All eight species and two hybrids of sturgeon were clearly delineated when the K value was set to 8, and the mean Q value exceeded 0.887. Each species formed a distinct group. Notably, hybrid groups 9 and 10 were identified as belonging to the *A. baerii* lineage.

### 3.4. The Hybrid Sturgeons Were Successfully Separated from Their Parents by FCA 

**Figure 3:**
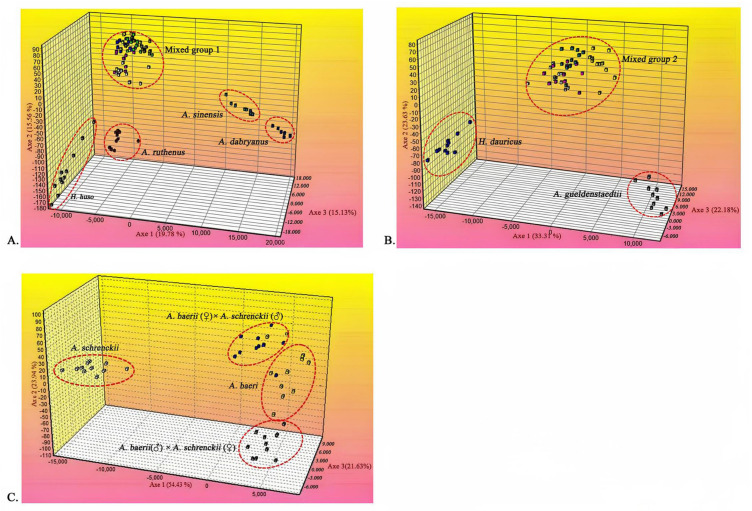
Factorial correspondence analysis (FCA) results. (**A**) FCA results for eight pure species and two hybrids of sturgeon. (1) *H. huso*; (2) *A. sinensis*; (3) *A. dabryanus*; (4) *A. ruthenus*; (5) a mixed group of six sturgeon species. (**B**) FCA results for four pure species and two hybrids of sturgeon. (1) *H. dauricus*; (2) *A. gueldenstaedtii*; (3) a mixed group of four sturgeon species. (**C**) FCA results for two pure species and two hybrids of sturgeon. (1) *A. baerii*; (2) *A. baerii* (♀) × *A. schrenckii* (♂); (3) *A. schrenckii*; (4) *A. baerii* (♂) × *A. schrenckii* (♀).

To further delineate the species of these samples, we genotyped 100 samples from eight sturgeon species and two hybrids, and performed clustering using FCA. The results showed that all the samples were grouped into five main clusters, each corresponding to one of the sturgeon species (Figure 3A). Specifically, cluster 1 represented *H. huso*, cluster 2 represented *A. sinensis*, cluster 3 represented *A. dabryanus*, and cluster 4 represented *A. ruthenus*. Conversely, the remaining six sturgeon species were grouped together in cluster 5, where they could not be differentiated. Notably, the most informative axis, the first axis, accounted for 19.78% of the total genetic variation, while the second and third axes contributed 15.56% and 15.13% of the divergent information, respectively. To further identify the species within cluster 5, an additional round of FCA was conducted using only samples from this cluster. Consequently, the six species were divided into three clusters (Figure 3B), with the information content of the three axes being 33.31%, 23.63%, and 22.18%, respectively. Cluster 1 corresponded to *H. dauricus*, and cluster 2 corresponded to *A. gueldenstaedtii*. However, the remaining four sturgeon species, including *A. baeri*, *A. schrenckii*, the orthogonal hybrid, and reciprocal hybrids, were grouped into cluster 3 due to their close genetic relationships, rendering them indistinguishable. To address the indistinguishability within cluster 3, another round of FPC analysis was conducted using the samples from this mixed cluster. The individuals were then divided into four clusters (Figure 3C): cluster 1 represented *A. baerii*, cluster 2 represented *A. baerii* (♀) × *A. schrenckii* (♂), cluster 3 represented *A. schrenckii*, and cluster 4 represented *A. baerii* (♂) × *A. schrenckii* (♀). The information content of the three axes was 54.43%, 23.94%, and 21.63%, respectively. These findings suggest that multiple rounds of FCA based on microsatellite loci can effectively identify different sturgeon species, even in cases where genetic relationships are closely intertwined.

## 4. Discussion

Research focusing on hybrid sturgeon derived from *A. baerii* and *A. schrenckii* primarily concentrates on aspects such as growth performance, fertility, stress resistance, and immunity [24,25,26]. Molecular markers exhibit significant advantages, as they reflect specific DNA fragments that indicate genetic differences within an organism’s genome, and reveal essential genetic variations among species [12]. Havelka et al. employed ddRAD sequencing to identify dinucleotide SNPs specific to *A. baerii* and *A. gueldenstaedtii*, and designed molecular markers capable of distinguishing these two species from other sturgeon species [12]. Havelka et al. utilized ddRAD to pinpoint specific dinucleotide SNPs of *A. ruthenus* and *H. Huso*, and developed molecular markers for identifying sterlet, beluga, and bester (sterlet × beluga) [13]. Boscari et al. amplified two intron sequences of the nuclear-coding gene Ribosomal Protein L8 (RPL8) from individuals of *H. dauricus* and *A. schrenckii*, and compared the sequencing results to discover specific mutations in the introns [14]. Yan et al. performed whole-genome resequencing of five sturgeon species, obtained species-specific InDel-based nucleotide sequences for *H. dauricus*, and developed methods for the rapid identification of Kaluga sturgeon germplasm [15]. Mitochondrial DNA barcoding is a species molecular marking technique that identifies species by analyzing the DNA sequence of a target gene. It has been widely used for molecular identification of species due to its high efficiency, precision, and global applicability [27]. When using DNA barcoding for species classification and identification, gene sequences with sufficient variation information, easy amplification, and a fragment that is characterized by species specificity and diversity are needed [28,29]. The COI gene encodes part of the terminal oxidase in the mitochondrial DNA, and is suitable for distinguishing and identifying genetic closely related species due to its abundant synonymous nucleic variations. Consequently, it is often used as a standardized tool for species classification and identification based on mitochondrial DNA barcoding. Pure sturgeon species such as *A. baerii*, *A. gueldenstaedtii*, *A. dabryanus*, *A. sinensis*, and *P. spathula* and the maternal parent of a hybrid offspring can all be identified through COI analysis [30,31,32,33]. In this study, the mitochondrial COI gene could successfully discriminate *A. baerii*, *A. schrenckii*, and their hybrid offspring from other sturgeon, but it could not distinguish the offspring from their female parents. This might be caused by the maternally inherited characteristics of mitochondrial DNA.

To address this issue, microsatellite markers were applied for further identification. Microsatellite DNA markers (SSRs) consist of tandem repeats of one to six nucleotides. SSR markers have demonstrated their effectiveness as molecular markers for species identification and genetic introgression analysis due to their high resolution, rapid detection capabilities, genetic stability, and codominant inheritance [34,35]. SSRs have been extensively utilized in population genetic studies and species identification of sturgeon species, including *A. gueldenstaedtii*, *A. sturio*, *A. transmontanus*, *A. stellatus*, *A. ruthenus*, *A. schrenckii*, *A. baerii*, and *H. huso* [36,37,38,39]. In this study, the structure analysis based on microsatellite loci obtained limited resolution among the eight species and two hybrids of sturgeons, especially for *A. baerii*, *A. schrenckii*, and their reciprocal hybrids. This limitation may be attributed to a certain degree of gene infiltration among different sturgeon species. Gene infiltration can enrich the gene pool and enhance genetic diversity; it may also lead to variations in genetic structure and germplasm confusion. The observed genetic infiltration in sturgeons might be a consequence of uncontrolled breeding practices and inadequate farm supervision. Factorial correspondence analysis (FCA) successfully divided the 100 samples from eight pure species and two hybrid sturgeons into 10 clusters. FCA progressively distinguishes different populations based on the proximity of their genetic relationships, starting from distant to close relationships, ultimately achieving the differentiation and identification of all populations. Dudu et al. [37] previously utilized FCA based on eight microsatellite loci to successfully discriminate pure species and hybrid sturgeons, including *H. huso*, *A. stellatus*, *A. ruthenus*, and *A. gueldenstaedti*, sourced from the lower Danube River. The findings of this study align with previous research, confirming the applicability of FCA for identifying sturgeon species and their hybrids.

Consequently, a variety of mitochondrial DNA genes, such as COI, Cyt b, 16S rRNA, and ND genes, are used as marker genes for species identification. Among them, the COI gene stands out due to its relatively conserved structure and substantial interspecific variation, providing abundant phylogenetic information. When using the COI gene for species identification, species exhibiting smaller genetic distances (typically, intraspecific genetic distances less than 0.03) or minimal differences in barcoding sequences (e.g., intraspecific sequence similarity differences in vertebrates less than 0.02, with interspecific differences exceeding 0.05) are generally considered to belong to the same species [27]. In this study, the genetic distance between *A. sinensis* and *A. dabryanus* was found to be 0.0034, significantly lower than that observed between *A. sinensis* and other sturgeon species. The phylogenetic analysis further corroborated the genetic similarity between *A. sinensis* and *A. dabryanus*. Zhang et al. previously reported a close genetic relationship between *A. sinensis* and *A. dabryanus*, which may be attributed to their overlapping distribution and extremely similar morphology, leading to *A. dabryanus* being previously regarded as a land-sealing ecotype of *A. sinensis* [40]. Alternatively, some studies propose that the small non-migrating sturgeon in the Yangtze River represents *A. dabryanus*, whereas the large migrating sturgeon is *A. sinensis* [41]. Considering the similar distribution of *A. sinensis* and *A. dabryanus*, the close genetic distance between them may suggest genetic exchange.

The findings of this study indicate that *A. baerii* and its orthogonal hybrid offspring *A. baerii* (♀) × *A. schrenckii* (♂) exhibited the highest genetic similarity, whereas *A. schrenckii* and its reciprocal hybrid progeny *A. schrenckii* (♀) × *A. baerii* (♂) demonstrated the closest genetic proximity. *Acipenser baerii* and *A. schrenckii* display varied mating patterns, and the probability of self-interbreeding and backcrossing in hybrid fish complicates the differentiation between species and hybrids. This study focused solely on distinguishing between *Acipenser baerii*, *A. schrenckii*, and their F1 hybrid sturgeon, neglecting the identification of self-interbreeding or backcrossing lines. In future research, we aim to address these limitations by utilizing a broader range of molecular markers and employing diverse methodologies to identify different reproductive lines of sturgeon.

## 5. Conclusions

This study established a method for identifying *A*. *baerii*, *A. schrenckii*, and their F1 hybrid sturgeon based on molecular markers of COI and SSR. This method not only facilitates the distinction of *A*. *baerii* and *A. schrenckii* from various other sturgeon species but also clearly differentiates the hybrid offspring from their parental species.

## Figures and Tables

**Table 1:** Information on six pairs of microsatellite loci used in this study.

Microsatellite Markers	Primer Sequences	Repetitive Sequence	Allele Size Range (bp)	Origin of the Primers
Ab3	F:CGCTTCACAGATGTCTCAGTCAGTT	CA	195–286	This study
	R:TCCTGAGTGGGGTTGTTCAATAAGA			
Ab5	F:TTTTCTCCACGAATGCCC	CA	107–175	This study
	R:TCAAAGTCAAGCCAAAGC			
As1	F: AACAAGCGACGAACAGTGTG	ATTG	242–301	This study
	R: CACAAATCGGACAGAAAGCA			
LS19	F: CATCTTAGCCGTCTGTGGTAC	TTG	116–153	Hu et al. [18]
	R: CAGGTCCCTAATACAATGGC			
HLJSX7	F: GAAAGGACACCAGCAGTG	GT	212–254	Hu et al. [18]
	R: AACCCATTAACAATTACAGC			
HLJSX30	F: GGGGAGAAAACTGGGGTAAA	CTAT	180–284	Hu et al. [18]
	R: CACGTGGATGCGAGAAATAC			

**Table 2:** Information about COI sequences of *A. schrenckii*, *A. baeri*, and their hybrids.

Species	T%	C%	A%	G%	C	V	Pi	S
*A. schrenckii*	26.0	30.5	22.5	21.0	659	25	3	22
*A. baeri* (♂) × *A. schrenckii* (♀)	25.8	30.7	22.2	21.3	662	1	0	1
*A. baeri*	26.7	30.2	22.8	20.3	661	0	0	0
*A. baeri* (♀) × *A. schrenckii* (♂)	26.7	30.2	22.8	20.3	660	0	0	0

C: conservative sites among the four breeds; V: variation sites among the four breeds; Pi: parsimony informative sites; S: single information sites.

**Table 3:** The mean intra- and interspecific divergences (Kimura 2-parameter distances) of *COI* among fifteen species and two hybrids of sturgeon.

Species	1	2	3	4	5	6	7	8	9	10	11	12	13	14	15	16	17
1		n/c	n/c	n/c	n/c	n/c	n/c	n/c	n/c	n/c	n/c	n/c	n/c	n/c	n/c	n/c	n/c
2	0.0034		n/c	n/c	n/c	n/c	n/c	n/c	n/c	n/c	n/c	n/c	n/c	n/c	n/c	n/c	n/c
3	0.0298	0.0298		n/c	n/c	n/c	n/c	n/c	n/c	n/c	n/c	n/c	n/c	n/c	n/c	n/c	n/c
4	0.0595	0.0595	0.0520		n/c	n/c	n/c	n/c	n/c	n/c	n/c	n/c	n/c	n/c	n/c	n/c	n/c
5	0.0501	0.0501	0.0427	0.0428		n/c	n/c	n/c	n/c	n/c	n/c	n/c	n/c	n/c	n/c	n/c	n/c
6	0.0652	0.0690	0.0575	0.0614	0.0209		n/c	n/c	n/c	n/c	n/c	n/c	n/c	n/c	n/c	n/c	n/c
7	0.0596	0.0633	0.0557	0.0595	0.0521	0.0596		n/c	n/c	n/c	n/c	n/c	n/c	n/c	n/c	n/c	n/c
8	0.0446	0.0446	0.0282	0.0617	0.0541	0.0654	0.0636		n/c	n/c	n/c	n/c	n/c	n/c	n/c	n/c	n/c
9	0.0464	0.0501	0.0426	0.0539	0.0354	0.0501	0.0521	0.0540		n/c	n/c	n/c	n/c	n/c	n/c	n/c	n/c
10	0.1298	0.1341	0.1274	0.1317	0.1391	0.1366	0.1280	0.1391	0.1392		n/c	n/c	n/c	n/c	n/c	n/c	n/c
11	0.1463	0.1463	0.1354	0.1403	0.1403	0.1514	0.1400	0.1449	0.1493	0.1041		n/c	n/c	n/c	n/c	n/c	n/c
12	0.1018	0.1018	0.0978	0.1040	0.1040	0.1124	0.1078	0.1021	0.1017	0.1928	0.1694		n/c	n/c	n/c	n/c	n/c
13	0.0996	0.0996	0.0956	0.1017	0.1017	0.1102	0.1055	0.0999	0.0994	0.1899	0.1667	0.0017		**0.0016**	n/c	n/c	n/c
14	0.0341	0.0341	0.0075	0.0470	0.0470	0.0657	0.0639	0.0360	0.0470	0.1325	0.1363	0.1046	0.1024		**0.0000**	n/c	n/c
15	0.0334	0.0334	0.0069	0.0463	0.0463	0.0650	0.0633	0.0354	0.0463	0.1339	0.1376	0.1019	0.0997	**0.0040**		**0.0000**	n/c
16	0.0483	0.0483	0.0445	0.0052	0.0052	0.0262	0.0539	0.0559	0.0372	0.1391	0.1360	0.1060	0.1038	**0.0488**	**0.0482**		**0.0000**
17	0.0483	0.0483	0.0445	0.0052	0.0052	0.0262	0.0539	0.0559	0.0372	0.1391	0.1360	0.1060	0.1038	**0.0488**	**0.0482**	**0.0000**	

Sturgeon species listed: (1) A. dabryanus; (2) A. sinensis; (3) A. transmontanus; (4) A. stellatus; (5) A. gueldenstaedti; (6) A. fulvescens; (7) A. ruthenus: (8) H. dauricus; (9) H. huso; (10) Psephurus gladius; (11) Polyodon spathula; (12) Scaphirhynchus albus; (13) S. platorynchus; (14) A. schrenckii; (15) A. schrenckii (♀) × A. baerii (♂); (16) A. baerii; (17) A. baerii (♀) × A. schrenckii (♂). Mean intra- and interspecific divergences are provided in percentages (%). The yellow background represents the hybrid sturgeon and their parental species. The green background represents the other sturgeon species utilized in this study. The intraspecific divergences are shown on the diagonal (in bold text), while the interspecific divergences are displayed below the diagonal. The standard errors of interspecific distance values among species are shown above the diagonal (n/c means that only one sample was available for analysis).

**Table 4:** Number of alleles detected from eight species and two hybrids of sturgeon by six microsatellite loci.

Specie	Number of Alleles (A)						
	As1	Ab3	Ab5	LS19	HLJSX07	HLJSX30	Average
*A. baerii*	6	4	5	6	5	6	5.3
*A. schrenckii*	1	4	3	3	4	4	3.2
*A. baerii* (♀) × *A. schrenckii* (♂)	6	5	4	5	3	4	4.5
*A. baerii* (♂) × *A. schrenckii* (♀)	5	7	4	4	3	5	4.7
*H. dauricus*	8	4	3	2	1	6	4.0
*H. huso*	6	6	5	6	2	4	4.8
*A. gueldenstaedtii*	5	4	3	6	3	3	4.0
*A. sinensis*	3	7	4	3	1	3	3.5
*A. dabryanus*	2	2	2	2	1	2	1.8
*A. ruthenus*	11	12	4	1	2	7	6.2
Total number	53	55	37	38	25	44	42.0

## Data Availability

The data that support the findings of this study are available in the additional files of this article.
